# Notes and News

**Published:** 1890-05-03

**Authors:** 


### Notes and News.

Collected and Communicated.

In gratitude for the deliverance from the perils of the voyage in the broken-down Inman liner, *City of Paris*, the passengers before landing subscribed £600 as a thank offering, and appointed a committee to decide upon its application. The committee have decided to hand over £400 to the Seamen's Hospital, Liverpool, for its general purposes, and to endow with the balance a bed in the same institution for sick American sailors.

Festival dinners were numerous last week. His Grace the Archbishop of Canterbury presided over the dinner in aid of King's College Hospital, and made an excellent speech; contributions amounting to £2,520 were announced. This amount was beaten by the Royal Hospital for Incurables, the secretary of which institution announced subscriptions amounting to £2,700 after their dinner. Be it mentioned in passing that the fair sex were prominent at this dinner. But the best sum of all was gained by the dinner at the Hotel Metropole, in aid of the German Hospital, the Duke of Cambridge in the chair. The contributions, connected with the festival, which included £200 from the Emperor of Germany, and £20 from the Duke of Cambridge, amounted to £3,540, which is £400 more than the amount last year.

The Duke of Westminster performed the simple ceremony of opening the West Ham Hospital, at Stratford. The hospital provides two wards for adults, fitted with twelve beds each, and there are two small wards for children, and a couple of isolation wards. The total cost of the institution was £7,000, exclusive of furniture, towards which a committee of ladies collected in a few months the sum of £1,063. The hospital replaces the West Ham, Stratford, and South Essex Dispensary, with which it is merged, and it is estimated that about £2,000 per annum will be required to maintain it efficiently. Subscriptions and donations were announced amounting to about £700, and included £220 odd from G.E.R. workmen, £50 from friendly societies, £100 by the Duke of Westminster, £250 by the Mayor of West Ham, and £50 annually for three years if nine others will give the same.

On Tuesday last the Duchess of Teck opened the annual United Sale of Work in aid of different Benevolent Institutions for Promoting Female Welfare. Amongst the institutions which had stalls were the Mary Wardell Home and the St. Marylebone Home for Incurables. One of the prettiest stalls was the flower stall, presided over by the Ladies Bernard.

The number of patients admitted last year to the St. Anne's Orphanage and Convalescent Hospital, Bridlington Quay, was 661, being an increase of 88 as compared with 1888. The revenue account shows a profit on the working of the Home of £289 15s. 5d., which, added to the balance brought forward from the previous year, £176 2s. 8d., makes a total of £465 28s. 1d.

Annual reports come pouring in. That of the Norfolk Hospital we have already noticed, but we are glad to see the Mayor presided when it was presented. The report from the North-Eastern Hospital for Children has a pretty wreath of snowdrops on its cover. Over 500 in-patients and 14,000 out were treated last year. The Central London Ophthalmic report tells a pleasant tale of ready assistance from Mrs. Frank Snoad and other friends. The report of the Great Northern Central Hospital we dealt with at the time of the annual meeting. There remains the Westminster Hospital report, which tells of 2,572 in-patients and 25,031 out-patients treated, an income of £9,290, and an expenditure of £13,376. New students last year numbered 23. Nearly 300 convalescent patients were helped by the Samaritan Fund.

Now that Succi, the Italian fasting man, is attracting universal attention it may be interesting to recall a case of total abstinence from food for forty days, which occurred more than two centuries ago. In the winter of 1684 a certain Isaac Henry Stiphont, of Haarlem, was confined in a lunatic asylum. At this date he was forty years old, and although born of an insane mother had learned a handicraft, married, and conducted himself like other people until, in the previous autumn, he quarrelled with his brother-in-law, and in a scuffle accidentally broke the man's leg, when the fear of falling into the hands of justice drove him mad. He had been in the asylum a few months when he suddenly took it into his head that he was the Messiah, and resolved to fast forty days and forty nights. Accordingly on December 6th he began to abstain from all food, and continued to do so until January 15th, 1685. During all this time he took no sustenance whatever; nothing passed his lips but an occasional sip of water for the purpose of cleansing his mouth. If a little broth or brandy was put into the water he discovered the addition instantly, and thrust the cup away untasted. Every effort was made to persuade or compel him to take food. It was even sought to influence him by the pretended apparition of an angel, who brought to him the express command of God that he should eat. He does not appear to have doubted the reality of the visitation, but continued to declare that it was the will of his Heavenly Father that he should fast forty days and forty nights. Stiphont had been a smoker before the commencement of his fast, and continued the daily use of tobacco during the whole time of his abstinence from food. The case had excited great interest, and when the fast was ended the doctors desired the man to take some medicine to stimulate the action of the stomach. He refused, and would only take fish and a special soup to be prepared by his wife. So singular an occurrence made a great noise at the time. Some people ascribed it to a miracle, others to the combined effect of madness and tobacco. A madman, it was said, could endure a temperature that froze his companions, so if insanity made a man impervious to cold, why should it not render him insensible to hunger? Also that the wild hordes of Canada were known, during times of scarcity, to exist for weeks upon water and tobacco, so why should not Stiphont, the civilized, do the same by the help of his madness?

The following vacancies are announced this week, the amount of the salary in each case being given after the name of the institution :—

**Resident Medical Officers.**

Brompton Cancer Hospital, House Surgeon—£60, board, etc.
Brompton Cancer Hospital, Registrar—£50, board, etc.
Royal South Hants Infirmary, Asst. House Surgeon—Board, etc.
York Dispensary, Medical Officer—£130, rooms, etc.
West Ham Hospital, Assistant House Surgeon—£30, board, etc.
Limpley Stoke Hydropathic, Medical Officer—£120, board, etc.
Swansea Hospital, Medical Officer—£100, board, etc.
Wandsworth Infirmary, Junior Assistant Medical Officer—£10, board, etc.

**Non-Resident Medical Officers.**

Salford Royal Hospital, Honorary Medical Officer.
Plymouth Hospital, three Honorary Assistant Surgeons.
Harrow Local Board, Health Officer—£50.
New Abbey Parochial Board, Medical Officer—£50.
Strathdon Parochial Board, Medical Officer—£65.

---

Amongst the pictures at the New Gallery of special interest to our readers are portraits of Dr. James Cagney, and of the two sons of Dr. W. Herringham. A picture by Edward Clifford of Father Damien's arrival at the leper settlement seems more allegorical than real; the head of Father Damien is fine enough, but the little dumpty figures of the lepers in the background are out of proportion. In the same room is a picture called "Consultation," representing two sisters of St. Vincent de Paul giving advice to a small patient. The anatomy is good on the whole, though one clever portrait is marred by a very dropsical and badly-drawn foot.

---

The bazaar this week in aid of the new Hospital for Women was a very grand affair, and offered such a feast of amusement as must have satisfied all comers. The dolls alone numbered some 15,000, but only about twenty of these were in nursing costume, and the prize was taken by Miss F. H. Tomson, while Nurse Florina and Nurse Armstrong secured honourable mention. Mrs. Scharlieb presided over a gorgeous Indian temple, which was excellently wrought out, and Mrs. Garrett Anderson was to the fore in all departments. Actresses and authors had sent autographs and photographs, and some of the best singers of the day helped at the concerts. Lewis Morris wrote an ode for the occasion, from which we quote the following :—

"To woman the Creator's hand has given
To tend the poor lives racked with hopeless pain,
And with a gracious ministry of Heaven,
To watch till vanished health revives again."

---

We have pleasure in announcing that her Royal Highness the Princess Beatrice has consented to open a bazaar for the Samaritan Free Hospital for Women and Children on Tuesday, May 6th, at three p.m., which will be held in the Portman Rooms, Baker Street, London, W., on May 6th, 7th, and 8th. The object of this bazaar is for the purpose of providing the additional furniture required, which will cost about £1,500, and of helping to raise the £10,000 still needed to pay for the building. The stalls have been named after spring flowers, and will be held as follows: "Lily of the Valley," by the Countess Cowley, assisted by her Grace the Duchess of Wellington and Lady Eva Wellesley; "Daffodils," by the Lady Knutsford and Mrs. A. J. Lewis; "Laburnum," by Lady Sophia Cecil; "Primroses," by the Dowager Lady Westbury; "Pink and White May," by the Hon. Mrs. Wm. Napier; "Heartsease," by the Duchess of Abercorn; "Lilacs," by Mrs. Montague Thorold, assisted by the Marchioness of Hertford, the Ladies Seymour, the Ladies Godolphin Osborne, and the Hon. Lady Thorold.

In the House of Lords, on Monday, it was resolved, on the motion of Lord Sandhurst, "That a Select Committee be appointed to inquire with regard to all hospitals and provident and other public dispensaries and charitable institutions within the metropolitan area for the care and treatment of the sick poor, which possessed real property or invested personal property, in the nature of endowment, of a permanent or temporary nature, and to receive, if the committee thought fit, evidence tendered by the authorities of voluntary institutions for like purposes, or with their consent, in relation to such institutions; and, further, to inquire and report what amount of accommodation for the sick was provided by rate, and as to the management thereof; and that the witnesses before the said Select Committee be examined on oath." We are glad to see Lord Sandhurst took care to say he approached the subject in no spirit of opposition to the hospitals. He knew the great straits in which they were placed, and what he wished to do was to lay bare all the facts, to strengthen worthy institutions, and, by taking the best advice, to suggest, if possible, some organization which, by the co-operation of different institutions, might render the enormous resources of charity and poor law more useful than at present. The following peers form the committee: The Archbishop of Canterbury, Earl Cadogan, the Earl of Winchelsea and Nottingham, the Earl of Lauderdale, Earl Spencer, Earl Cathcart, the Earl of Kimberley, Lord De La Zouche, Lord Clifford of Chudleigh, Lord Sandhurst, the Earl of Erne, Lord Lamington, the Earl of Arran, Lord Monkswell, and Lord Thring.

